# Effect of race and ethnicity on utilization and outcomes of assisted reproductive technology in the USA

**DOI:** 10.1186/s12958-017-0262-5

**Published:** 2017-06-08

**Authors:** Alice J. Shapiro, Sarah K. Darmon, David H. Barad, David F. Albertini, Norbert Gleicher, Vitaly A. Kushnir

**Affiliations:** 1 Center for Human Reproduction, 21 East 69th Street, New York, NY 10021 USA; 2 Department of Obstetrics, Gynecology & Women’s Health, Rutgers New Jersey Medical School, Newark, NJ USA; 3 Foundation for Reproductive Medicine, New York, NY USA; 4 Stem Cell Biology and Molecular Embryology Laboratory, The Rockefeller University, New York, NY USA; 5 Department of Obstetrics and Gynecology, University of Vienna School of Medicine, Vienna, Austria; 6 Department of Obstetrics and Gynecology, Wake Forest School of Medicine, Winston-Salem, NC USA

**Keywords:** Race, Ethnicity, Disparity, Third-party assisted reproduction technology

## Abstract

**Background:**

The purpose of this study was to determine the utilization and live birth rates of assisted reproductive technology (ART) modalities among various racial and ethnic groups in recent years.

**Methods:**

We reviewed ART data reported to the Society for Assisted Reproductive Technologies Clinic Outcome Reporting System (SART CORS) for autologous ART and third-party ART (3ART) cycles which involved donor oocytes, sperm, embryos and gestational carrier, performed in the U.S. between 2004 and 2013. To gauge demand by various racial/ethnic groups for ART services, we examined fertility rates and demographics of the entire U.S. birth cohort over the same time interval.

**Results:**

Of 1,132,844 autologous ART cycles 335,462 resulted in a live birth (29.6%). An additional, 217,030 3ART cycles resulted in 86,063 live births (39.7%). Hispanic and Black women demonstrated high fertility and lower utilization rates of autologous ART and 3ART. Caucasian and Asian women exhibited lower fertility rates and higher autologous ART and 3ART utilization. Autologous ART resulted in higher live birth rates among Caucasian and Hispanic women and lower rates among Asian and especially Black women. 3ART improved live birth rates in all races/ethnicities, though Black women experienced lower live birth rates with most modalities. Spontaneous abortion rates were higher among Black women following autologous ART and some 3ART modalities than those among Caucasian women.

**Conclusion:**

Utilization of ART is inversely related to fertility rates. Autologous ART produces lower live birth rates among Asian and Black women. 3ART results in relatively low live birth rates among Black women.

**Trial registration:**

SART CORS #57, Registered 5/14/2015

## Background

Racial and ethnic disparities in the United States health care system have been well established, [1] with members of racial and ethnic minority groups generally demonstrating poorer health outcomes than non-Hispanic White patients. Differences persist even when adjusting for disease severity, insurance coverage, age and income [1]. Such disparities have also been reported in association with assisted reproductive technology (ART), using autologous patient gametes and womb [2].

It has been reported that non-Hispanic Black women have 24–38% lower live birth rates than non-Hispanic Whites in fresh non-donor ART cycles, even when adjusting for confounders [3]. They also reportedly experience more spontaneous abortions, defined as pregnancy loss prior to 20 weeks. This difference may be attributable in part to a higher prevalence of uterine leiomyomas in the non-Hispanic Black population [4], as they are also significantly more likely to have uterine factor infertility than non-Hispanic White women [5]. In contrast, non-Hispanic White women uniformly demonstrate higer live birth rates using ART than all other racial/ethnic groups [3].

Asian women are also less likely to achieve successful pregnancy and live birth using autologous ART than non-Hispanic White women, [6–9] with the likelihood of achieving clinical pregnancy being reduced by approximately 14% and likelihood of achieving live birth reduced by 10% [10]. This difference persists even when controlling for confounders such as age, infertility diagnosis and number of embryos transferred [6, 9].

Race and ethnicity, therefore, appear to be important predictors of ART outcome. However, which race/ethnicity-dependent factors are responsible for these differences in ART outcomes has remained controversial. Some authors have suggested cultural and socioeconomic differences [3–5], while we previously suggested a genetic predisposition characterized by differences in the distribution of *FMR1* gene mutations. So-called *low FMR1* mutations (CGG _*n*<26_) have been associated with poorer ART outcomes [11, 12] and are found at much higher prevalence in women of African descent than in Caucasian and especially Asian women [13, 14]. In contrast, Asian women have the highest number of so-called *high FMR1* mutations (CGG _*n*>34_) [13, 14] and both *low* and *high* FMR1 mutations have been associated with low functional ovarian reserve [15–17]. Other potential mutations, mostly affecting the androgen metabolism in women of African descent, have also been suggested as a possible etiology [18].

3ART provides an option for parenthood in patients who cannot achieve it via autologous ART with autologous gametes and/or womb. 3ART encompasses use of donor oocytes, donor sperm, donor embryos, or gestational carriers and any combination thereof. As women increasingly delay childbearing into their late 30s and 40s, reproduction with autologous oocytes becomes less successful. To achieve reproductive goals at later ages, oocyte donation has become an integral and growing part of ART, now accounting for approximately 12% of all ART cycles [19].

3ART modalities offer relatively high birth rates, and thus contribute disproportionately to the total ART birth cohort, currently representing 1.6% of all newborns in the U.S. [20]. We found that in recent years 3ART was utilized in 16.1% of all ART cycles and contributed to 20.9% of all live born infants after ART [21]. The primary goal of this investigation was to determine utilization and live birth rates of ART and 3ART among racial and ethnic groups in comparison to those groups respective contributions to the entire U.S. birth cohort over the past decade. This analysis refines understanding of the impact of biological and social factors which underlie infertility and determine utilization and outcomes of its treatment via ART.

## Methods

This study was approved by the IRB of the Center for Human Reproduction and by the Research Committee of the Society for Assisted Reproduction (SART). De-identified patient data were obtained from the SART Clinical Outcome Reporting System (CORS). The SART CORS database contains more than 90% of all ART cycles performed in the U.S. Data is voluntarily reported by most U.S. ART centers. ART cycles which do not involve in vitro fertilization, such as intrauterine insemination, are not included in this registry. Data is verified by SART and reported to the Centers for Disease Control and Prevention in compliance with the Fertility Clinic Success Rate and Certification Act of 1992 (Public Law 102–493). The data are validated annually with select centers having on-site visits for chart review. Ten out of 11 data fields selected for validation were recently found to have discrepancy rates of ≤5% [20].

All autologous ART and 3ART cycles performed between 2004 and 2013 were analyzed. A total of 1,132,844 autologous ART cycles, which resulted in 335,462 live births, were thus available for analysis. In addition, we analyzed 217,030 3ART cycles which resulted in 86,063 live births. We excluded 36.9% of total reported cycles which did not report race/ethnicity. Cycles which did not report race/ethnicity decreased from 42.7% in 2004 to 35.6% in 2013. The cycles which did not report race/ethinicty were similary distributed between autologous and 3ART (36.8% vs. 37.9%).

To gauge baseline fertility rates in each racial/ethnic group, we referred to demographic data from birth certificates of the entire U.S. birth cohort for women aged 15 to 44 over the same time interval published in the National Vital Statistics Report [22].

Utilization of autologous ART and 3ART was calculated based on age and race/ethnicity of the intended mother. Live birth rates were calculated for each ART modality per total number of fresh and frozen ART cycles. Women were categorized into the following racial/ethnic groups: non-Hispanic White (White/Caucasian), non-Hispanic Black (Black/African), Hispanic, Asian/Pacific Islander (Asian). We excluded women categorized as Other race (consisting of American Indian, Alaskan Native and mixed race) from analysis. Data was insufficient for detailed analysis of American Indian and Alaskan Native women who account for approximately 1% of the US birth cohort but only 0.2% of women utilizing ART, though we were able to report demographics for this group. People of mixed race were excluded due to differences in source data handling between U.S. Vital Statistics which uses an algorithm to assign mixed race to a single race category and SART which does not.

Statistical analyses were conducted using SAS version 9.4 (SAS Institute Inc., Cary, NC). Live birth, clinical pregnancy and spontaneous abortion rates were evaluated in a modified Poisson regression using generalized estimating equation (GEE) model to control for patients with multiple cycles which utilized non-Hispanic White as the race reference group and adjusted for age of the intended mother and oocyte donor. All statistical tests utilized were 2-sided, with α level of 0.05 defined as significant.

## Results

### Utilization of ART

Table 1 demonstrates that utilization of autologous ART and 3ART was highest among non-Hispanic White and lowest among non-Hispanic Black women in the major race/ethnic groups. When considering the utilization of ART in comparison to the proportion of women of each race and ethnicity in the entire birth cohort, the table also shows that non-Hispanic White and Asian women are over-represented, while non-Hispanic Black and Hispanic women are under-represented. American Indian/Alaska Native women also had very low utilization of ART.

**Table 1:** Demographics of the US birth cohort and of women undergoing ART 2004–2013

	Non-Hispanic White	Non-Hispanic Black	Asian/Pacific Islander	Hispanic	Other		Missing
					American Indian/Alaska Native	Multi-Race	
Autologous ART cycles	515,263	50,298	87,845	58,494	1462	2966	416,516
3ART cycles	101,454	8764	13,635	9715	216	988	82,258
Race Distribution							
US Birth Cohort (40,942,262 birth)	54.3%	14.6%	6.1%	23.8%	1.1%	-	-
Autologous ART	72.2%	7.1%	12.3%	8.2%	0.2%	-	-
3ART	75.8%	6.6%	10.2%	7.3%	0.2%	-	-
Oocyte Donation	74.5%	6.7%	11.8%	6.9%	0.2%	-	-
Sperm Donation	76.7%	8.6%	6.6%	8.0%	0.2%	-	-
Embryo Donation	88.1%	3.7%	3.4%	4.7%	0.1%	-	-
Gestational Carrier	76.4%	3.9%	10.5%	9.1%	0.1%	-	-
Multiple 3ART	78.6%	4.9%	7.9%	8.5%	0.1%	-	-
Mean Age ± SD							
US First Birth, 2013	26.8	23.9	29.4	24.0	22.9	-	-
Autologous ART	35.4 ± 4.7	36.3 ± 4.9	36.0 ± 4.5	35.8 ± 4.9	35.6 ± 4.6	36.1 ± 4.8	36.1 ± 4.8
3ART	40.1 ± 5.5	41.6 ± 5.4	41.3 ± 5.6	40.1 ± 5.9	40.1 ± 5.5	41.3 ± 5.1	40.6 ± 5.3
Age Distribution							
Autologous ART							
<35	46.4%	38.0%	40.4%	42.0%	47.3%	40.7%	40.6%
35–37	22.5%	22.2%	24.2%	22.7%	20.6%	21.8%	22.7%
38–40	18.5%	22.0%	20.6%	20.4%	18.3%	21.1%	20.6%
41–42	4.6%	5.7%	5.2%	5.2%	4.9%	5.6%	5.5%
>42	8.0%	12.2%	9.7%	9.7%	9.0%	10.8%	10.6%
3ART							
< 35	16.2%	9.8%	12.5%	17.7%	18.1%	9.7%	13.6%
35–37	12.9%	9.7%	11.5%	12.7%	13.4%	11.1%	12.1%
38–40	18.7%	17.7%	17.1%	17.5%	17.1%	17.8%	18.6%
41–42	8.2%	8.2%	6.6%	7.4%	9.3%	6.8%	8.2%
> 42	44.0%	54.7%	52.3%	44.8%	42.1%	54.6%	47.6%
Infertility Diagnosis							
Male Infertility	36.4%	31.9%	33.6%	36.2%	31.4%	32.1%	32.2%
Endometriosis	12.0%	7.2%	10.0%	10.1%	9.4%	8.5%	9.9%
Polycystic Ovaries	15.3%	11.0%	13.8%	13.2%	19.2%	11.1%	12.5%
Diminished Ovarian Reserve	27.6%	27.3%	31.0%	24.8%	23.1%	36.6%	27.4%
Tubal	14.0%	35.5%	14.1%	24.2%	22.2%	16.0%	16.0%
Uterine	4.7%	12.9%	5.2%	5.3%	4.9%	6.4%	6.4%
Unexplained	11.2%	6.4%	12.7%	8.0%	8.5%	10.4%	13.8%
Other	15.0%	13.2%	14.1%	15.6%	16.8%	16.8%	15.1%

Hispanic, non-Hispanic Black, and American Indian/Alaska Native women experienced their first delivery at younger ages than non-Hispanic White, and especially Asian women, who in 2013 delivered their first child at a mean age of 29.4 years. In the general population, the age of first birth increased in 2013 compared to 2004 by 0.6 years for non-Hispanic White, 1.2 years for non-Hispanic Black, 1.0 years for Asian and by 0.9 years for Hispanic women.

Non-Hispanic Black women utilizing ART and 3ART, were marginally older than women of other races undergoing the procedure with a mean age of 37.1, compared to ages of non-Hispanic White, Asian and Hispanic women, 36.2, 36.8, 36.4 respectively. Between 2004 and 2013 among all patients undergoing ART the mean age increased by 0.3 years.

Table 1 shows distribution of infertility diagnosis among all women utilizing ART. Non-Hispanic Black women have a higher prevalence of tubal factor and uterine factor infertility than non-Hispanic White women. Further stratification of this data showed that women utilizing 3ART had a relatively higher prevalence of diagnosis of diminished ovarian reserve and uterine factor infertility as recently described [21].

Table 1 also summarizes autologous ART and 3ART cycles in each racial/ethnic group: non-Hispanic White women demonstrated high utilization of all ART modalities but especially of the 3ART modality embryo donation. Asian women demonstrated relatively high utilization of oocyte donation and of gestational carriers, while non-Hispanic Black women utilized sperm donation relatively frequently but were least likely to utilize gestational carriers among the major groups.

**Fig. 1:**
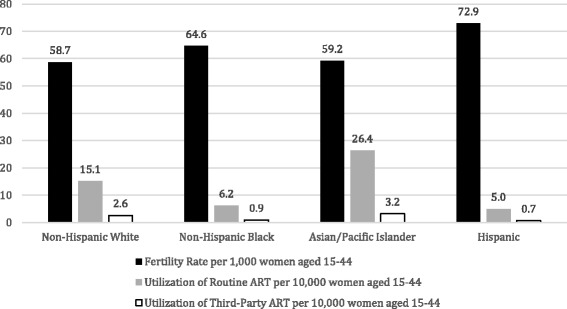
Fertility rate and utilization of ART in 2013 based on race and ethnicity of the intended mother

Figure 1 demonstrates that fertility rates per 1000 women aged 15–44 in 2013 were relatively high among Hispanic and non-Hispanic Black women and lower among non-Hispanic White and Asian women. This figure also shows that utilization of ART and 3ART per 10,000 women aged 15–44 mirrors fertility rates in the general population, with highest utilization seen among Asian and non-Hispanic White women and lowest among Hispanic and non-Hispanic Black women.

**Fig. 2:**
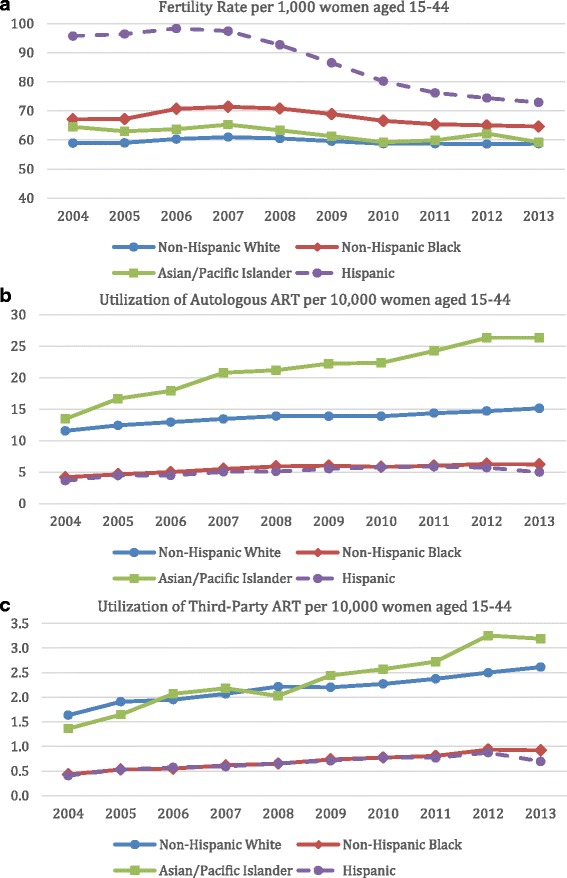
Fertility rates (**a**) and utilization of autologous ART (**b**) and third-party ART (**c**) over time, based on race and ethnicity of the intended mother

Figure 2 demonstrates fertility rates and utilization of autologous ART and 3ART for the four major racial/ethnic groups. Figure 2a shows that after 2007 fertility rates declined in all groups, but the decline was most pronounced among Hispanic women, though they remained higher than those of other groups. Figure 2b and c demonstrate gradually increasing utilization of autologous ART and 3ART respectively among all groups, but especially pronounced among Asian women.

**Fig. 3:**
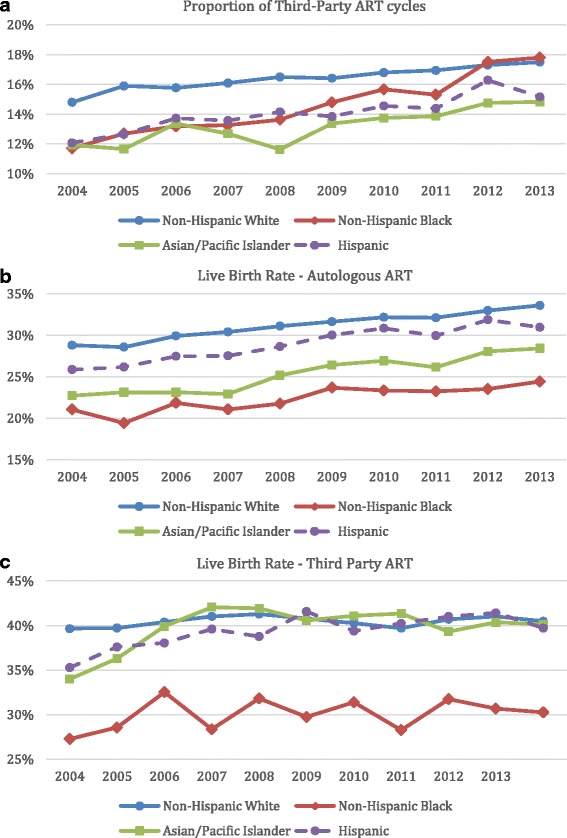
Proportion of third-party ART cycles (**a**), live birth rates in autologous ART (**b**) and third-party ART cycles (**c**) over time, based on race and ethnicity of the intended mother

Figure 3a shows that utilization of 3ART has increased for all racial and ethnic groups. This figure also shows that in the most recent years the proportion of 3ART cycles performed in non-Hispanic Black women has increased more rapidly than in other groups, slightly surpassing the proportion in non-Hispanic White women.

### Live birth rates

Figure 3b shows that while unadjusted live birth rates in autologous ART cycles have increased over time for all racial and ethnic groups, the rates were highest among non-Hispanic White women and lowest among non-Hispanic Black women for the duration of the study. Figure 3c shows that while unadjusted live birth rates for all 3ART cycles have slightly increased over time, they were lowest among non-Hispanic Black women.

**Fig. 4:**
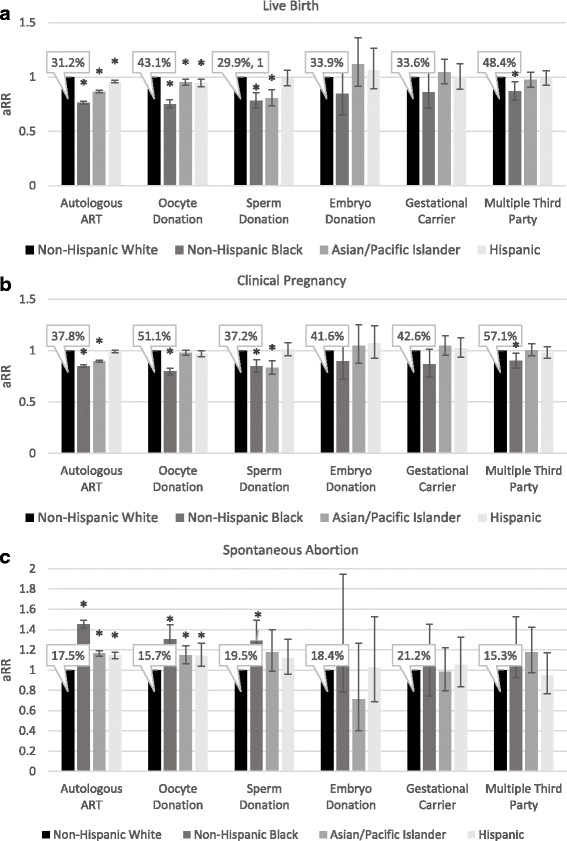
Adjusted relative risk for live birth rates (**a**), pregnancy rates (**b**), and spontaneous abortion rates (**c**) for each ART modality, stratified by race and ethnicity of the intended mother. Non-Hispanic White women were the reference group for all comparisons, data is adjusted for age of the intended mother and oocyte donor. A generalized estimating equation (GEE) model was used to controlled for patients with multiple cycles. Bars represent 95% confidence intervals. Percentages indicate live birth rates for each treatment modality among Non-Hispanic White women. * indicates *P*-value <0.05

More detailed information including adjusted relative risks of live birth are presented in Fig. 4a. Live birth rates for each ART modality among non-Hispanic White women are indicated; adjusted relative risks for all other racial/ethnic groups are presented in relation to this reference group. In autologous ART, non-Hispanic White women achieved the highest (31.2%) and non-Hispanic Black women the lowest (22.5%) live birth rates, while Asian women fell in between (25.8%). Hispanic women, in contrast, almost reached live birth rates of non-Hispanic White women at 29.3%.

Figure 4a also shows that among 3ART cycles, in all racial/ethnic groups, highest live birth rates were achieved with use of multiple-3ART modalities, followed by oocyte donation. 3ART with sperm donation produced highest live birth rates among non-Hispanic White and Hispanic women and significantly lower rates among Asian and non-Hispanic Black women. Embryo donation and gestational carrier outcomes were similar when comparing non-Hispanic White women to each of the other racial/ethnic groups. Non-Hispanic Black women achieved significantly lower live birth rates with multiple 3ART modalities in comparison to non-Hispanic White women.

Lower live birth rates observed among non-Hispanic Black women with autologous ART and some 3ART modalities such as oocyte donation are attributable to both significantly lower clinical pregnancy rates (*P* < 0.0001 for both, Fig. 4b) and higher spontaneous abortion rates as noted in detail below and in Fig. 4c.

### Spontaneous abortion rates

Figure 4c shows absolute risk of spontaneous abortion for non-Hispanic White women for each ART modality as well as adjusted relative risks for other race/ethnic groups. In pregnancies conceived with autologous ART spontaneous abortion rates were lowest at 17.5% among non-Hispanic White women and highest at 26.6% among non-Hispanic Black women. Spontaneous abortion rates were lowest at 15.7% of pregnancies conceived with donor oocytes among non-Hispanic White women and highest at 21.2% of those among non-Hispanic Black women. Spontaneous abortion also affected 19.5% of 3ART pregnancies conceived with donor sperm among non-Hispanic White women and 26.5% of those among non-Hispanic Black women. Figure 4c indicates that all the above noted differences in spontaneous abortion rates remained significant after statistical adjustment. Spontaneous abortion rates with embryo donation, gestational carrier, and multiple 3ART modalities did not vary statistically among racial and ethnic groups.

## Discussion

Over the past three decades, fertility rates have increased among women in their 30s and 40s as more women are delaying pregnancy to older reproductive ages [22]. Simultaneously, utilization of autologous ART and 3ART has grown particularly rapidly among older women [21]. Additionally, an increase in the number of same-sex couples and of single men and women seeking parenthood, likely, also contributed to increased use of 3ART.

The data presented here suggest that in comparison to their respective contribution to the general U.S. birth cohort, non-Hispanic White and Asian women are relatively over-represented and non-Hispanic Black and Hispanic women are under-represented among those pursuing autologous ART and 3ART. These disparities may be to some degree attributable to differences in access to care and economic, educational, as well as, cultural factors [23, 24]. However, the data also suggests that because of differences in inherent fertility rates and mean ages at first birth, clinical demand for fertility services, varies among racial/ethnic groups. It is reasonable to conclude that increased demand leads to increased utilization of assisted fertility services.

The data clearly show that utilization of ART mirrors fertility rates and mean age at first birth. With relatively low fertility rates and first births at older ages, non-Hispanic White and Asian women demonstrate relatively high demand for infertility treatment. In contrast, non-Hispanic Black and Hispanic women, with relatively high fertility rates and younger ages at first delivery have lower demand and therefore lower utilization of infertility treatments. The same pattern can be seen among American Indian/Alaska Native women who have the earliest age at first birth, and lowest relative utilization of ART and 3ART. Socioeconomic barriers likely amplify this phenomenon among those minority women who desire fertility services but are unable to utilize them due to lack of access, affordability or social acceptance in their communities.

Like prior investigators we noted a decline in fertility rates in the general population after year 2007, the period following the last economic recession (Fig. 2a) [25]. Declines in fertility rates in the general population were especially pronounced among minorities, which has previously been attributed to disproportionate effects of the recession on incomes of minorities. The significant decline in Hispanic fertility rates after 2007 may also reflect lower levels of immigration into the U.S., in the mid-2000s, as first-generation Hispanic immigrants tend to have larger families than subsequent generations [25]. Additionally, increased educational attainment by Hispanic and other minority women may also play a role in declining fertility rates [26].

Multiple prior studies reported that success rates of autologous ART varied among different racial/ethnic groups [10, 20, 27]. This study is the first to also confirm these outcome differences in 3ART. Though non-Hispanic Black women undergoing autologous ART and 3ART in this study were slightly older than non-Hispanic White women, their significantly lower live birth and higher spontaneous abortion rates persisted after adjustment for age of patient and oocyte donor in our statistical models. Our data, therefore, add further credence to the hypothesis that race/ethnicity of the patient may be a truly independent predictor of success with infertility treatment.

While we did not have access to race demographics of gamete donors, it is likely that non-Hispanic Black women would preferentially select donors of the same race. Prior studies have found significantly lower ovarian reserve based on measurements of antimullerian hormone among Hispanic, non-Hispanic Black and Asian women in comparison to non-Hispanic White women [28, 29]. It is likely that multiple genetic and environmental factors which affect the ovarian reserve and the rate of reproductive aging, therefore, affect chances of success with both autologous ART and 3ART [30]. As noted earlier, Black women carry significantly more *low FMR1* mutations than Caucasian and especially Asian women, [13, 14] which have been associated with lower functional ovarian reserve and poorer IVF outcomes [11]. Interestingly, Asian oocyte donors demonstrate low functional ovarian reserve more frequently than Caucasian donors [31]. They also have the highest prevalence of *high FMR1* mutations, [14, 32] which also have been associated with low functional ovarian reserve [16, 17]. The *FMR1* gene may, therefore, be one important genetic factor that predisposes some Asian, and especially non-Hispanic Black women to poorer ART outcomes.

Women of African descent are found to carry more frequent mutations, which impair androgen metabolism, especially the conversion of dehydroepiandrosterone (DHEA) to testosterone [18]. Since adequate testosterone levels are now widely considered essential for normal folliculogenesis, [33] those mutations could also play a crucial role in ART outcomes in non-Hispanic Black women.

Previously noted higher incidence of myomas in non-Hispanic Black women, [4] would suggest that they would be more likely to utilize gestational carriers. Surprisingly, we found that not to be the case, and, thereby confirmed a previous report [34]. The significant cost of a gestational carrier may explain this finding.

Interestingly, non-Hispanic Black women demonstrate relatively young age at first birth and relatively high fertility rates in the general population. They, however, represented the oldest patient group pursuing both autologous ART and 3ART. These observations suggest that there is a subpopulation of non-Hispanic Black women who delay fertility until older ages and subsequently encounter infertility which is relatively difficult to overcome with both autologous ART and 3ART. These are important observations that require further inquiry since non-Hispanic Black women, especially at older ages, experience the highest maternal pregnancy risks, including mortality [35]. Early identifications of such women by providers of family planning services could offer potential opportunities for early interventions, which may improve both maternal and fetal outcomes [36, 37].

This study is limited by the lack of required reporting by ART clinics to SART of racial/ethnic data in a significant percentage (36.9%) of all ART cycles. We found that the proportion of cycles which did not report racial/ethnic data have somewhat decreased in more recent years, this highlights the importance of reporting race and ethnicity information by individual practices when submitting data to SART. We found that demographics for this group of patients were very similar to those with reported race/ethnicity and are evenly distributed between 3ART and autologous ART (Table 1). This suggests that the data is missing at random and is therefore unlikely to be a major source of bias in this study. We report demographics for patients with mixed or missing race in Table 1, however, due to differences in source data handling in the Vital Statistics report and SART dataset, described in the methods section we decided to exclude these groups from additional analysis. Race and ethnicity reported by patients to fertility centers and subsequently to SART is typically self-determined based on patients’ interpretation which may be influenced by multiple factors, including life experiences, and therefore may not represent true genetic differences. Since race/ethnicity categories are broad, women with very different backgrounds may be classified together; for example, Indian and Japanese women may be categorized as Asian. Additionally, demographics of gamete donors is not currently tracked by SART. This study is strengthened by its large sample size with over 90% of ART clinics in the United States contributing to data reporting. To our knowledge this is the first study to examine utilization and outcomes of 3ART based on race and ethnicity.

## Conclusions

Utilization of ART is inversely related to fertility rates in the general population. Our findings suggest that lower utilization of ART among Black and Hispanic women is primarily due to lower demand rather than barriers to access. Autologous ART produces lower live birth rates among Asian and Black women. 3ART results in relatively low live birth rates among non-Hispanic Black women, suggesting presence of biological and social factor which are more difficult in to overcome with modern infertility treatments than those present in other groups. Mounting evidence indicates that race and ethnicity strongly influence utilization and outcomes of ART. Focused research in this area may, therefore, help to better tailor counseling and fertility treatments to individual patients.

## Abbreviations

ART: Assisted reproductive technology
3ART: Third-party assisted reproductive technology
SART CORS: Society for Assisted Reproductive Technologies Clinic Outcome Reporting System
